# Potential for Prebiotic Stabilized *Cornus mas* L. Lyophilized Extract in the Prophylaxis of Diabetes Mellitus in Streptozotocin Diabetic Rats

**DOI:** 10.3390/antiox11020380

**Published:** 2022-02-14

**Authors:** Szymon Sip, Daria Szymanowska, Justyna Chanaj-Kaczmarek, Krystyna Skalicka-Woźniak, Barbara Budzyńska, Olga Wronikowska-Denysiuk, Tymoteusz Słowik, Piotr Szulc, Judyta Cielecka-Piontek

**Affiliations:** 1 Department of Pharmacognosy, Poznan University of Medical Sciences, Rokietnicka 3, 60-806 Poznan, Poland; szymonsip@ump.edu.pl (S.S.); justyna.chanaj-kaczmarek@ump.edu.pl (J.C.-K.); 2 Department of Biotechnology and Food Microbiology, Poznan University of Life Sciences, 31 Wojska Polskiego Street, 60-634 Poznan, Poland; darszy@up.poznan.pl; 3 Department of Natural Products Chemistry, Medical University of Lublin, 1 Chodźki Street, 20-093 Lublin, Poland; krystyna.skalicka-wozniak@umlub.pl; 4 Independent Laboratory of Behavioral Studies, Medical University of Lublin, 20-093 Lublin, Poland; barbara.budzynska@umlub.pl (B.B.); olga.wronikowska-denysiuk@umlub.pl (O.W.-D.); 5 Experimental Medicine Center, Medical University of Lublin, Jaczewskiego 8D, 20-090 Lublin, Poland; tymoteusz.slowik@o2.pl; 6 Department of Agronomy, Poznań University of Life Sciences, Dojazd 11, 60-632 Poznan, Poland; piotr.szulc@up.poznan.pl

**Keywords:** *Cornus mas*, diabetes mellitus, microbiome, prebiotics, natural compounds, civilization disease

## Abstract

As a systemic disease, diabetes mellitus (DM) is characterized by the disruption of many glucose metabolic pathways. Therefore, it seems critical to study new therapies to support treatment to develop therapeutic systems that can operate across a broad metabolic spectrum. The current state of knowledge indicates an essential role of the gut microbiota in the development and course of the disease. *Cornus mas* fruits have demonstrated a rich biological activity profile and potential for application in the treatment of DM. As part of a preliminary analysis, the activity of four cultivars of *Cornus mas* fruits was analyzed. The cultivar *Wydubieckij* was selected as having the highest activity in in vitro conditions for further prebiotic system preparation. The study aimed to develop a unique therapeutic system based, first of all, on the mechanism of α-glucosidase inhibition and the antioxidant effect resulting from the activity of the plant extract used, combined with the prebiotic effect of inulin. The obtained system was characterized in vitro in terms of antioxidant activity and enzyme inhibition capacity, and was then tested on diabetic rats. The study was coupled with an analysis of changes in the intestinal microflora. The system of prebiotic stabilized *Cornus mas* L. lyophilized extract with inulin offers valuable support for the prophylaxis and treatment of DM.

## 1. Introduction

Raw materials of plant origin are increasingly used to treat and support diseases of civilization, particularly diabetes mellitus (DM) [1,2]. Due to the complexity of the causes and effects of DM, most patients, in addition to polytherapy, also use dietary supplements to support glycemic control [3,4]. Plant raw materials are a rich source of active substances with high therapeutic potential. In the case of such a complex disease as DM, they will not fully replace conventional pharmacotherapy, but they create the possibility of reducing the doses used or reducing the number of drugs used in pharmacotherapy to control glycemia [5,6]. The primary method of reducing blood sugar levels is diet modification, which is always the first step in implementing a therapeutic program. Providing adequate nutrients in the diet is vital for two reasons—they can lead to a direct reduction in blood sugar and stimulate the composition of the gut microbiome, which is essential for inhibiting the development of DM.

*Cornus mas* L. (also known as Cornelian cherry) is one of the most valuable fruit plants with hypoglycemic potential that belongs to the Cornaceae family. The *Cornus* genus consists of about 50 species of Cornelian cherry, occurring naturally in the temperate climate zone. The Cornelian cherry can mainly be found as a shrub or a small tree, growing up to 9 m high. It grows in central and south-western Europe, to the Caucasus and western Asia [7]. The fruits of Cornelian cherry can be oval-shaped, bottle-shaped, or pear-shaped, and range in color from dark red to yellow [8].

*Cornus mas* fruits have a strong inhibitory effect on exocrine enzymes responsible for breaking down complex carbohydrates (α-amylase and α-glucosidase) into easily digestible simple sugars [9,10]. The inhibition of these enzymes limits the absorption of simple sugars, contributing to the hypoglycemic effect, without interfering with intracellular metabolic pathways. The early stages of DM development are characterized by an increased production of oxygen radicals which translates into more significant oxidative stress. This causes damage to pancreatic β-cells, leading to insufficient insulin production. The excessive production of oxygen radicals persists throughout the development of the disease, contributing to DM progression over time. The increased amount of oxygen radicals causes the glycation of proteins, determining the main metabolic changes resulting from DM [11]. Due to high polyphenol content, *Cornus mas* fruits show high antioxidant potential, decreasing oxidative stress, and reducing and preventing the cascade of adverse symptoms arising in the course of the disease [12,13]. *Cornus mas* fruits also contain iridoids, which may affect insulin metabolism. In particular, loganin and loganic acid are important due to their properties that reduce intraocular pressure [14], protect against autoimmune disease [15], help reduce inflammation [16], and protect blood vessels [17]. In the course of DM, patients often struggle with the occurrence of glaucoma, which is the result of damage to the optic nerve caused by high intraocular pressure. Moreover, in the course of the disease, problems are often observed with the venous system, as a secondary consequence of excess weight and little physical effort, causing swelling of the limbs and lymphatic effusions [18,19]. An essential aspect of DM is extensive inflammation resulting from improper carbohydrate metabolism [20,21]. For this reason, it seems crucial to incorporate consideration of the influence of iridoids in research. Their presence in the plant raw material directly affects the glucose metabolic pathway and can eliminate potential secondary symptoms occurring as part of a multi-level response.

Previous studies indicate the potential of *Cornus mas* fruit to inhibit lipase. In vitro studies have confirmed the beneficial effects of *Cornus mas* fruit on the lipid profile while suppressing inflammation of blood vessels [22]. Studies in rats with hypercholesterolemia have stimulated glutathione production with antioxidant properties [23]. Numerous in vivo and in vitro studies have been carried out which consider the high potential of *Cornus mas* fruit in treating type 2 DM. The fruit has also been shown to have a beneficial effect on the expression of peroxisome proliferator-activated receptor (PPAR) genes that act as transcription factors for genes involved in metabolism and inflammation [10,15,24,25,26,27].

Current knowledge indicates the significant importance of the gut microbiota in preventing DM and the effectiveness of implemented treatment [28]. Maintaining the intestinal microbiome’s correct composition protects against potential pathogens and maintains the intestinal epithelium’s integrity. In addition, studies have demonstrated an essential role for a healthy microbiome in glucose tolerance by enhancing tissue response to insulin [29]. This role is attributed to short-chain fatty acids formed in the fermentation of complex carbohydrates [30,31,32]. A critical aspect resulting from the intestinal microbiota composition is the pH of the environment in which they occur. The excessive development of specific microorganisms, and the metabolism that they lead to, may change the pH of the gastrointestinal tract. Such a change will promote the growth of selected microorganisms; reducing the diversity of the intestinal ecosystem will also aggravate the problem. Particularly important for the proper functioning of the intestinal microflora is the presence of bacteria with pro-health properties, mainly belonging to the genera *Lactobacillus* and *Bifidobacterium*, and the appropriate supply of prebiotic substances ensuring their proper growth [32,33,34,35].

The research carried out was based on the assumption that it is necessary to combine the ability to reduce the absorption of simple sugars and the antioxidant activity produced by the plant extract, with prebiotic activity, that can alleviate the course of DM over a long period, through the use of inulin as a prebiotic carrier. The research undertaken aimed to assess the possibility of using Cornelian cherry extract in supporting DM treatment, informed by the latest literature reports indicating the intertwined development and course of the disease with the state of the intestinal microbiota. The research began with the preparation of extracts that were standardized for selected active compounds. A sequence of tests using in vitro models was then performed to assess the α-glucosidase inhibition capacity and the antioxidant potential using 2,2-diphenyl-1-picrylhydrazyl (DPPH) and ferric reducing antioxidant power assay (FRAP), leading to selection of a variety from which to prepare a prebiotic pre-formulation. As a result of the research conducted, an inulin-based pre-formulation was obtained which was further tested in vivo in rats. The effect on the change in the intestinal microflora in the tested animals was assessed.

## 2. Materials and Methods

### 2.1. Materials and Instruments

Standard compounds used in the HPLC analysis including loganic acid (≥95%), delphinidin 3-*O*-glucoside (≥95.0%), cyanidin-3-*O*-glucoside (≥95.0%), and pelargonidin 3-*O*-glucoside (≥95.0%) were supplied by Sigma-Aldrich, St. Louis, MO, USA.

Reagents used in the biological activity studies, including α-D-glucopyranoside (PNPG), α-glucosidase from *Saccharomyces cerevisiae* (Type I, lyophilized powder, ≥10 units/mg protein), acarbose, 2,2-diphenyl-1-picrylhydrazyl, TPTZ (2,4,6-tripyridyl-S-triazine) and iron (III) chloride hexahydrate (FeCl_3_·6H_2_O) were supplied by Sigma-Aldrich, St. Louis, MO, USA. Folin–Ciocalteu reagent was supplied by Fischer Chemicals (Fisher Scientific, Waltham, MA, USA).

For the microbiological studies the following were assessed: The total number of bacteria (5% sheep blood agar, bioMerieux, Craponne, France), number of *Bifidobacterium* (agar BSM, Sigma-Merck; St. Louis, MO, USA), *Enterococcus* and *Escherichia coli* bacteria (chromogenic CPS, bioMerieux, Marcy-l’Étoile, France), including the potentially pathogenic *E. coli* form Biovare (ENDO, Heipha), anaerobic *Clostridium* (TSC, Biocorp, Issoire, France), lactobacilli (*Lactobacillus* (MRS agar, Oxoid, Basingstoke, UK)), other *Enterobacteriaceae* (*Enterobacter* spp., *Proteus* spp.) and of the genus *Pseudomonas*, and the number of yeast-like fungi of the genus *Candida* (CHROMagar Candida, CHROMagar Company, Springfield, NJ, USA).

The organic solvent evaporation process was carried out using rotavapor BÜCHI B-490. The lyophilization process was carried out in a lyophilizer (Heto PowerDry PL3000) Freeze Dryer (Thermo Scientific, Waltham, MA, USA). Qualitative and quantitative research was carried out using the ultra-high performance liquid chromatograph DionexUltiMate 3000 coupled a with DionexUltiMate 3000 RS Diode Array Detector with the computer program DionexChromeleon Version 7.12.1478. The extraction was carried out using an ultrasonic bath (Elmasonic S180H, Singen, Germany). For the biological tests the following were used: a centrifuge (Nüve NF800, Ankara, Turkey), a plate reader (Multiskan GO (Thermo Scientific), and a laboratory incubator (MaxQ 4450, Thermo Scientific). The water content of the raw material was determined using a moisture analyzer (OHAUS MB120). To measure weight, a Radwag AS 220.X2 (Radom, Poland) analytical balance was used throughout the study.

### 2.2. Plant Material

Ripe *Cornus mas* L. fruits were collected from the “Szynsad” fruit farm in Dąbrówka Nowa, Błędów, Mazowieckie, Poland (51°47′01″ N 20°43′04″ E) in 2018. *Cornus mas* fruit came from the third year of plantation cultivation. Fruit of *Cornus mas* of four cultivars were obtained: *Bolestraszycki*, *Florianka*, *Słowianin*, and *Wydubieckij*. A selection of fruit was made with all fruits containing blemishes and traces of possible penetration by insect larvae removed. Fully ripe, red fruits were obtained for the research.

### 2.3. Corni fructus Extract Preparation

The fruits were thoroughly washed to remove any mechanical and chemical impurities that could affect further testing. The next step was to remove the stones for easier processing in later stages of the study. The fruits were wiped dry, frozen at −22 °C, and then freeze-dried.

Freeze-dried *Cornus mas* fruit was sieved through a 0.5 mm sieve. An amount of 10 g of fruit was weighed into a 100 mL volumetric flask on an analytical balance, supplemented with distilled water until proportional, incubated for 1 h in an ultrasonic bath at 40 °C, and then centrifuged at 9000 r/min for 10 min to produce a clear supernatant. The obtained stock solution, with a concentration of 0.1 g of freeze-dried fruit/mL, was used to investigate the polyphenol, loganic acid (LA) and anthocyanin content, antioxidant activity, and α-glucosidase inhibition. HPLC analysis samples were also filtered through a 0.2 mL filter (Merck-Millipore Burlington, MA, USA) to remove mechanical impurities

### 2.4. Phytochemical Analysis of Aqueous Extracts

#### 2.4.1. Determination of Anthocyanin Content

The HPLC gradient method, coupled with a UV-Vis detector, enabled the qualitative and quantitative determination of three anthocyanins in the *Corni fructus* extracts (Table S1). The analyses were carried out using a Zorbax Eclipse Plus C18 column (4.6 mm × 100 mm; 3.5 µm), the mobile phase containing 0.1% formic acid (A), and acetonitrile (B). The gradient developed to fit the requirements of the method assumed changes in the mobile phase according to the scheme: 0–45 min B = 10–20%, 45–60 min B = 20–30%, 60–70 min B = 30–40%. The method then included a 10 min re-equilibration period to establish the mobile phase equilibrium column relative to the initial mobile phase ratio. The phase flow was set at 0.3 mL/min, injection for all test samples was 10 μL, and the detection was performed at 520 nm. The anthocyanin retention times in this analytical method were, respectively, delphinidin 3-*O*-glucoside (6.16 min), cyanidin-3-*O*-glucoside (8.93 min), and pelargonidin 3-*O*-glucoside (12.15 min). Identity confirmation was based on differences in retention times, coupled with the compound’s spectrum obtained relative to the standard during the analysis. The results are presented as µg/mL, where the weight refers to the dry weight of the freeze-dried fruit used to prepare the extract.

#### 2.4.2. Determination of Loganic Acid Content

The HPLC gradient method, coupled with DAD detector, allowed the qualitative and quantitative determination of loganic acid in water extracts from *Corni fructus (*Figure S1 and Table S2). The determinations were carried out using a Zorbax SB-C18 column, Rapid Resolution 4.6 mm × 100 mm 3,5—Micron; the mobile phase contained 0.1% acetic acid (A) and methanol (B). The gradient developed for the needs of the method assumed changes in the mobile phase according to the scheme: 0–4 min B = 1%, 4–18 min B = 1–12%, 18–22 min B = 12–20%, 22–35 min B = 20–35%, 35–40 min B = 30–95%, 40–50 min B = 95%. The method considers 10 min re-equilibration time to determine the phase equilibrium column relative to the initial injection phase. The phase flow was set to level 1 mL/min, injection for all test samples was 5 μL, the detection was carried out at 240 nm. The LA retention time in this analytical method was 23.5 min. The results are presented as µg/mL, where the weight refers to the dry weight of the freeze-dried fruit used to prepare the extract.

#### 2.4.3. Determination of Total Phenolic Content (TPC)

TPC was determined using the Folin–Ciocalteu method with minor modifications. A 50 µL plant extract solution diluted 10 times was mixed with 50 µL of Folin-Ciocalteu reagent (F.-C.) and 100 µL distilled water. The mixture was pre-incubated for 5 min at 37 °C with shaking at 100 rpm. Then 100 µL 20% Na_2_CO_3_ aq. solution was added and incubated for 30 min at 37 °C with shaking at 100 rpm. The absorbance was read at 750 nm against the blank sample (water instead of the extract) in sixplicate. TPC was expressed as mg of gallic acid equivalent (GAE) per g of lyophilized *Corni fructus* utilizing a standard curve of gallic acid (y = 9.7183x − 0.2776; R^2^ = 0.9984) in the concentration range 0.06–0.2 mg/mL [36]. The content of TPC in the tested extract was calculated following the standard curve for gallic acid. The curve used to calculate the TPC content in the form of gallic acid as a conversion factor is presented in the Supplementary Materials (Figure S5).

### 2.5. In Vitro Activity of Corni fructus Extracts

#### 2.5.1. α-Glucosidase Inhibitory Assay

A spectrophotometric method with minor modifications was used to determine the inhibition of α-glucosidase by the *Corni fructus* water extracts [37]. Briefly, 50 μL of sample solution (80–160 µg/mL *Wydubieckij*, 480–560 µg/mL *Słowianin*, 500–580 µg/mL *Florianka* and 470–550 µg/mL *Bolestraszycki*) or acarbose (positive control, 1–5 mg/mL) in different concentrations, 50 μL of 0.1 M phosphate buffer (pH 6.8) and 30 µL α-glucosidase solution (1.0 U/mL) was pre-incubated in 96 well plates at 37 °C for 15 min. Next, 20 μL of 5 mM p-nitrophenyl-α-D-glucopyranoside (pNPG) solution in a 0.1 M phosphate buffer (pH 6.8) was added and incubated at 37 °C for 20 min. The reaction was terminated by adding 100 µL of sodium carbonate (0.2 M) into the mixture. The absorbance of the liberated p-nitrophenol was measured at 405 nm. The absorbance of enzyme solution, but without plant extracts/acarbose, served as the control with total enzyme activity. The absorbance in the absence of the enzyme was used as the blind control. The enzyme inhibition rate, expressed as a percentage of inhibition, was calculated using the following formula:

% inhibition activity = ((A_C_ − A_S_)/A_C_) × 100

where A_C_ is the absorbance of the control (100% enzyme activity), and A_S_ is the absorbance of the tested sample (*Corni fructus* water extract or acarbose). For the investigated extracts, two independent experiments were carried out in triplicate. Results were expressed as means ± S.D. The results are presented as µg/mL, where the weight refers to the dry weight of the freeze-dried fruit used to prepare the extract.

#### 2.5.2. DPPH Assay

The DPPH assay was effected according to Studzińska-Sroka et al., with modifications [38]. Briefly, 25 μL of extracts of *Corni fructus* dissolved in distilled water at different concentrations (0.25–8.0 mg/mL), was mixed with 175 μL of DPPH solution (3.9 mg in 50 mL of MeOH). The reaction mixture was shaken and incubated in the dark at room temperature for 30 min. The control contained 25 μL of distilled water and 175 μL of DPPH solution. Absorbance was measured at 517 nm. The inhibition of the DPPH radical by the sample was calculated according to the following formula:

DPPH scavenging activity (%) = ((A_C_ − A_S_)/A_C_) × 100%

where A_C_ is the absorbance of the control and A_S_ is the absorbance of the sample. The results are presented as µg/mL, where the weight refers to the dry weight of the freeze-dried fruit used to prepare the extract.

#### 2.5.3. FRAP Assay

Following Tiveron et al. (2012), the FRAP assay was performed with some modifications [39]. The stock solutions of FRAP reagent included 300 mM acetate buffer (pH 3.6), 10 mM TPTZ solution in 40 mM HCl, and 20 mM FeCl_3_·6H_2_O solution. The working FRAP solution was freshly prepared by mixing 25 mL of acetate buffer, 2.5 mL of TPTZ solution, and 2.5 mL of FeCl_3_·6H_2_O solution and then warmed at 37 °C before usage. Briefly, 25 μL of the tested extracts dissolved in distilled water at different concentrations (1.0–6.0 mg/mL) were mixed with 175 μL of FRAP solution, shaken, and incubated 37 °C for 30 min. in the dark condition. Then the absorbance was read at 593 nm. The results were expressed as the IC_0.5_, corresponding to the extract concentration required to produce a 0.5 O.D. value. The results are presented as µg/mL, where the weight refers to the dry weight of the freeze-dried fruit used to prepare the extract.

### 2.6. Preparation of Corni fructus Extract System with Prebiotic Carrier

An amount of 20 g of the freeze-dried, previously sieved, fruit of *Cornus mas Wydubieckij* variety was extracted twice with 250 mL of 70% ethanol for 60 min at 45 °C on an ultrasonic bath. The obtained extracts were concentrated below 50 °C under vacuum to dryness. The extract was standardized for TPC, loganic acid, and anthocyanin content (Table S3). Following this, dry extract was mixed with 200 mL of 10% inulin aqueous solution (an amount of 20 g of inulin was added to the dry extract), frozen, and then lyophilized. The process was carried out for 72 h to remove all water from the system; the automatic procedure programmed in the freeze dryer (Heto PowerDry 3000) was used. The resulting formulation was triturated in an agate mortar to obtain a homogeneous powder.

### 2.7. In Vivo Antidiabetic Activity of Prebiotic Stabilized Lyophilizate of Corni fructus

The experiments were carried out on 40 naive male Wistar rats (from the Center for Experimental Medicine of the Medical University of Lublin) weighing 200–250 g. The animals were kept under standard laboratory conditions (12 h light/dark cycle, room temperature 21 ± 1 °C) with free access to tap water and a laboratory chow (Agropol, Warsaw, Poland). Each experimental group consisted of 10 animals. All experiments were being conducted according to the National Institute of Health Guidelines for the Care and Use of Laboratory Animals and in accordance with the European Community Council Directive for the Care and Use of Laboratory Animals of 22 September 2010 (2010/63/E.U.), and were approved by the local ethics committee (96/2019).

Rats were randomly divided into four groups: the control group, STZ-treated group, control group treated with *Corni fructus* extract with inulin, and STZ-treated with *Corni fructus* extract with inulin. In the DM group, animals received an intraperitoneal injection of freshly prepared streptozotocin (STZ), singe injection, 60 mg/kg, in sterile sodium citrate buffer at pH 4.5 [40]. *Corni fructus* extract with inulin at a dose of 50 mg/kg m.c (dose based on the obtained pre-formulation containing inulin and plant extract suspended in it) was administered intragastrically for three weeks once daily. Fasting blood glucose was measured using an Accu-Check blood glucose meter (Roche, Pleasanton, CA, USA) on the 14th and 21st day from day 1 of the injection of STZ.

Bodyweight and blood glucose levels were monitored. Rats with blood glucose levels higher than 270 mg/dL (>15 mmol/L) were considered diabetic [41].

### 2.8. Quantitative and Qualitative Analysis of Microorganisms Present in the Gastrointestinal Tract

An amount of 0.2 g of feces was collected and placed in 2.0 mL 0.9% NaCl for bacteriological studies. The procedure was repeated until a dilution of 10^−6^ was obtained. A series of appropriate dilutions were inoculated in a volume of 50 μL and spread with a sterile loop on selectively differentiating and propagating media. The cultures were grown under conditions specific for a given group of microorganisms: *Bifidobacterium*, *Clostridium*—48 h, at 37 °C, in anaerobic conditions; *Lactobacillus* 48 h, at 37 °C, under microaerophilic conditions; *Enterococcus*, *Enterobacteriaceae* rods and *Proteus* rods—24 h, at 37 °C, under aerobic conditions.

For the qualitative and quantitative determination of fungi, 0.2 g of feces was collected and placed in 2 mL of trypsin solution with the addition of 25 µg of antibiotics (penicillin and streptomycin) to inhibit bacterial growth. The samples were placed at 37 °C for 15 min to digest food residues to prevent fungal growth. The samples were washed in a buffered physiological PBS solution, then inoculated on two fungal growth media, Sabouraud, or chloramphenicol (bioMerieux). Incubation was conducted at 37 °C for 48 h and room temperature (20 °C) for 48 h, respectively. This was to distinguish fungi from the environment from potentially pathogenic fungi. Then identification was made using *Candida* chromogenic medium. Mold fungi were identified based on direct preparation and a mycological key.

### 2.9. Statistical Analysis

Analysis of biological activity in vitro was performed in at least six replicates. Statistical analysis was performed using Statistica 13.3 software (StatSoft Poland, Krakow, Poland). The Shapiro–Wilk test was implemented to check data distribution normality and the Levene’s test assessed the equality of variances. Statistical significance was performed using a one-way analysis of variance (ANOVA) followed by the Bonferroni post hoc test. Measurements were considered significant at *p* < 0.05.

## 3. Results and Discussion

### 3.1. Quantitative and Qualitative Analysis of the Examined Extracts

The first stage of the research was the standardization of water extracts from lyophilized *Cornus mas* fruits from three Polish cultivars *Słowianin*, *Florianka*, *Bolestraszycki*, and one Ukrainian cultivar *Wydubieckij.* These were standardized for the content of anthocyanins, loganic acid, and polyphenols taking into account the water content of freeze-dried fruit (Table S4). 

**Figure 1:**
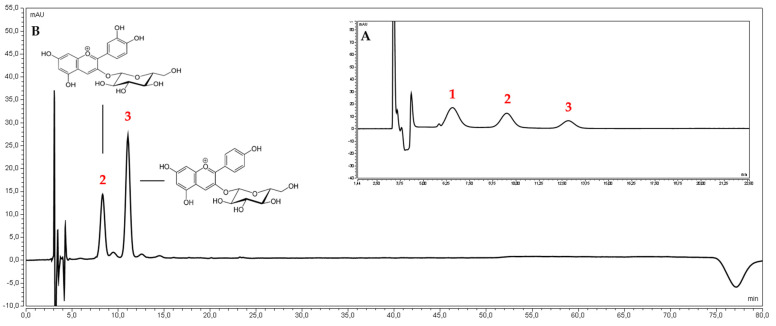
The chromatogram of the reference substances. (**A**) 1-delphinidin 3-*O*-glucoside, 2- cyanidin 3-*O*-glucoside, 3- pelargonidin 3-*O*-glucoside, and (**B**) water extract of *Corni fructus* cultivar *Wydubieckij*.

**Figure 2:**
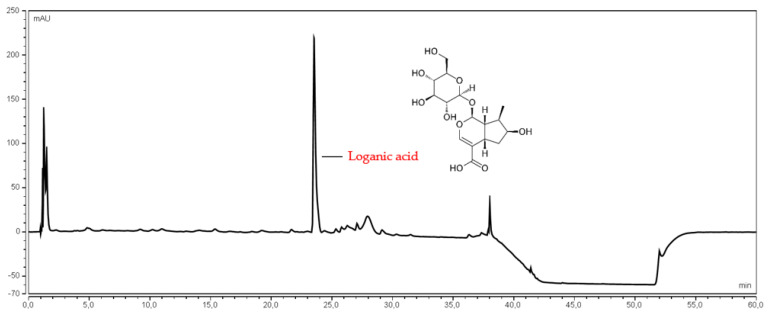
The chromatogram of loganic acid presented in the water extract of *Corni fructus* cultivar *Wydubieckij*.

Ultra-high performance liquid chromatographic methods, using a diode array detector (UHPLC-DAD) with gradient elution, were developed to determine anthocyanin 3-*O*-glucosides of pelargonidin, cyanidin, delphinidin, and loganic acid (Figure 1 and Figure 2). The UHPLC-DAD methods were validated according to the International Conference on Harmonization (ICHQ2) guidelines [42]. The validation parameters are presented in the Supplementary Materials (Tables S1 and S2). The peaks of studied compounds in the *Cornus mas* fruits water extracts were compared with the reference substances’ retention times and UV spectra.

Anthocyanin content depends on the cultivar, environmental factors and growth, and storage conditions [43]. Table 1 shows that the major anthocyanin in *Corni fructus* extracts for the cultivars *Bostraszycki*, *Florianka*, and *Słowianin* was pelargonidin 3-*O*-glucoside ranging from 2.21 ± 0.19 to 3.09 ± 0.25 mg/g. On the other hand, the *Corni fructus* cultivar *Wydubieckij* contained a similar amount of pelargonidin 3-*O*-glucoside and cyanidin 3-*O*-glucoside, which was found to be 0.10 ± 0.02 mg/g and 0.10 ± 0.00 mg/g, respectively. Delphinidin 3-*O*-glucoside was not found in any water extracts of *Cornus mas* fruit. Based on the literature, anthocyanins are usually extracted from plant material with an acidified organic solvent (mainly alcohols or alcohol-water mixtures) [44]. These solvents destroy the cell membranes of vacuoles, and dissolve and stabilize the anthocyanins [45]. However, pH reduction is not always necessary to increase these compounds’ extraction efficiency [43]. The previous studies reported comparable results when determining anthocyanin content [46,47,48]. Similarly, the *Corni fructus* cultivars *Bostraszycki*, *Florianka*, and *Słowianin* water extracts showed a comparable content of loganic acid; values were 5.38 ± 0.52, 4.71 ± 0.45, 4.96 ± 0.12, respectively. The lowest content was found in the *Wydubieckij* variety, 2.82 ± 0.27. According to the literature data, the concentration of loganic acid in different cultivars of *Corni fructus* ranged from 2.26 to 8.20 mg per g of dry fruit [49,50].

Additionally, the method described by Blainski et al. to determine the total phenolic content, with gallic acid as the standard, was used [36]. The highest phenolic compounds (16.03 ± 1.28 mg GAE/g) were found in the water extract from the *Corni fructus* cultivar *Wydubieckij*. The phenolic compounds concentration in other cultivars ranged from 7.34 ± 0.66 to 8.94 ± 0.83 mg GAE/g of lyophilized *Corni fructus* extracts (Table 1) [46].

### 3.2. In Vitro Activity

The antidiabetic properties of *Corni fructus* water extracts were evaluated for their ability to inhibit α-glucosidase, relevant to small intestinal glucose uptake (Table 2). Several reports have indicated the α-glucosidase inhibitory properties of plant extracts and isolated compounds [51,52,53].

**Figure 3:**
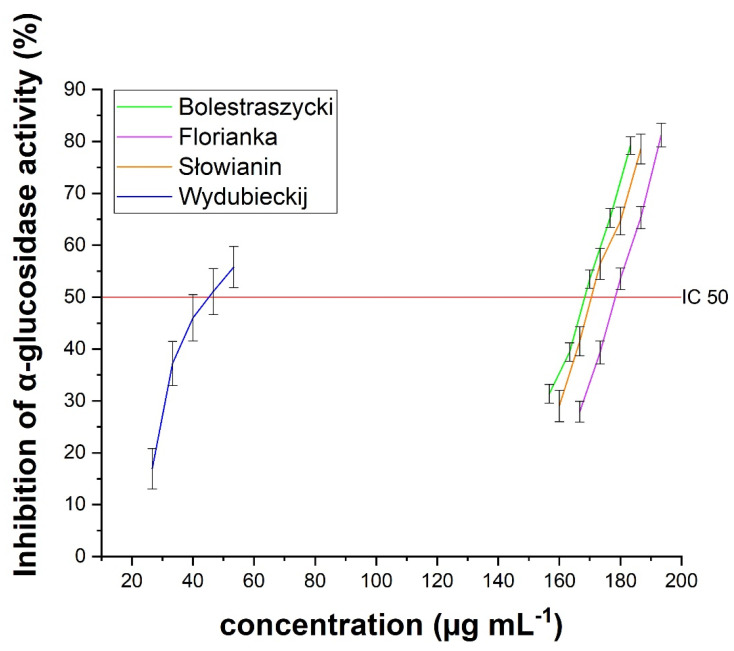
The α-glucosidase inhibitory activity of *Corni fructus* water extracts. Significance with *p* ≤ 0.05.

**Figure 4:**
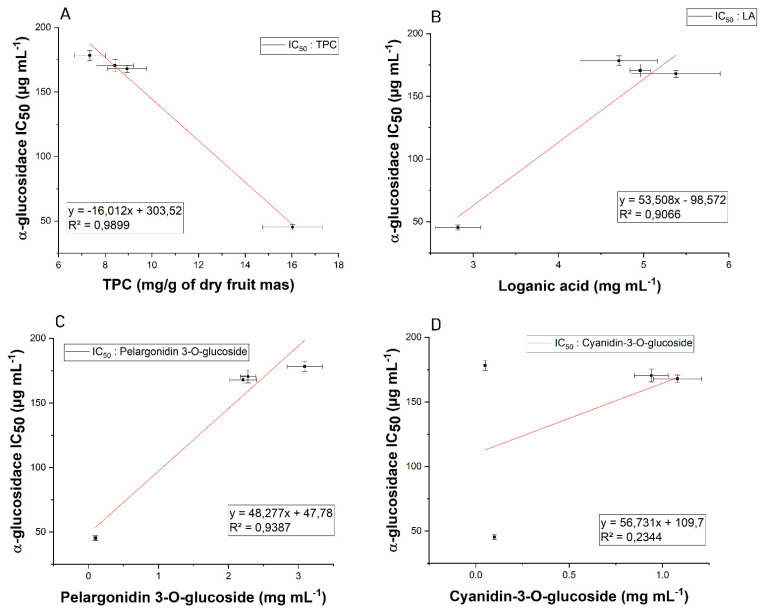
Correlation between α-glucosidase inhibitory activity and TPC (**A**) *, loganic acid (**B**) *, pelargonidin 3-*O*-glucoside (**C**) *, and cyanidin 3-*O*-glucoside (**D**) ** content (*—significance with *p* ≤ 0.05; **- significance with *p* > 0.05).

The water extracts of *Corni fructus* showed significant α-glucosidase inhibitory activity (Figure 3). The most potent inhibitory activity was observed for the *Wydubieckij* cultivar (IC_50_ = 45.23 µg/mL), which was 30 times more potent than the acarbose standard (IC_50_ = 1.22 mg/mL). The obtained results demonstrate the significant potential for using the studied extracts in support of the treatment of DM, based on the inhibition of the digestive enzyme α-glucosidase. In particular, the obtained results show higher activity for the tested extracts than acarbose, which is successfully used in medicine and typically exhibits an inhibitory effect on the enzyme. Among compounds capable of reducing blood glucose levels, phenolic compounds, mainly anthocyanins, and iridoids, should be mentioned [54]. The conducted studies showed a strong positive correlation between glucosidase inhibitory activity and TPC (R^2^ = 0.9899) (Figure 4), which indicates the effect of the synergistic action of several classes of phenolic compounds, such as phenolic acids (e.g., chlorogenic acid, gallic acid) and flavonoids (especially flavonols and flavan-3-ols, which are well-known as good glucose-lowering agents [55,56,57,58,59].

Moreover, the statistical analysis performed showed a significant (*p* < 0.05) negative correlation between the obtained concentration of loganic acid and pelargonidin 3-*O*-glucoside (R^2^ = 0.9066 and R^2^ = 0.9387, respectively) with the ability of the extract to inhibit α-glucosidase. The obtained results showed a decrease in the activity of the extract with an increasing amount of the indicated compounds. However, in the study of enzyme inhibition capacity, it was shown that higher extract concentrations showed a higher enzyme inhibition capacity. It can be concluded that the observed increasing inhibitory activity of the extract with increase in its concentration results from the presence of compounds that show much more potent enzymatic inhibition, which translates into effective increased activity. No statistically significant correlation was found between cyanidin 3-*O*-glucoside and α-glucosidase inhibition (R^2^ = 0.2344; *p* > 0.05).

**Figure 5:**
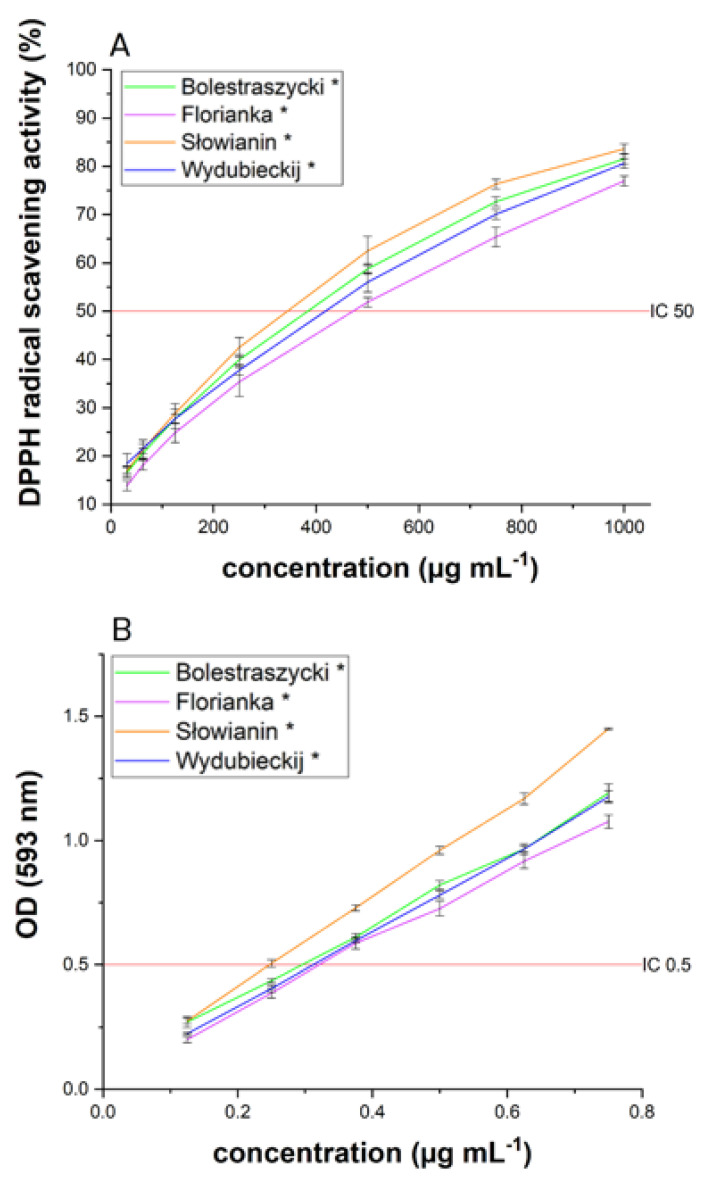
Antioxidant activity of *Corni fructus* water extracts by DPPH (**A**) and FRAP (**B**) assays. Data expressed as mean ± SD; * significance with *p* ≤ 0.05.

Oxidative stress results from an imbalance between systems producing radicals and neutralizing radicals, i.e., increased free radicals, reduced antioxidant defense activity, or both. Several studies have shown that diabetes is accompanied by increased free radical formation and reduced antioxidant capacity, which leads to loss of pancreatic β-cell function, aggravation of insulin resistance, and vascular complications [60,61]. The total antioxidant capacity of *Cornus mas* fruits water extracts was assessed by two spectrophotometric methods (DPPH and FRAP) that utilize the SET (single electron transfer) mechanism. Most natural antioxidants are multifunctional, so it is essential to carry out more than one type of antioxidant capacity measurement to cover different antioxidant activity mechanisms [62]. As shown in Figure 5 and Table 2, the most potent antioxidant activity was produced by water extract from the *Corni fructus* cultivar *Słowianin* with IC_50_ = 343.63 µg/mL and IC_0.5_ = 0.25 µg/mL, respectively, in the DPPH and FRAP assays. The other *Cornus mas* fruit cultivars showed comparable antioxidant activity, however slightly weaker than the *Słowianin* cultivar. The correlation coefficients between TPC and antioxidant activity were not significant (*p* > 0.05), suggesting that the free-radical scavenging activity of *Corni fructus* water extracts may be attributed to differences in the activity of phenols and other active compounds, such as ascorbic acid [63], monoterpenes (especially limonene) and iridoids [64,65]. On the other hand, the antioxidant potential for the entire extract, which was tested, is an exponential result of single reactions taking place in the single electron transfer process, which does not fully reflect the reactions taking place in vivo, providing only an estimate of the antioxidant capacity [66].

**Figure 6:**
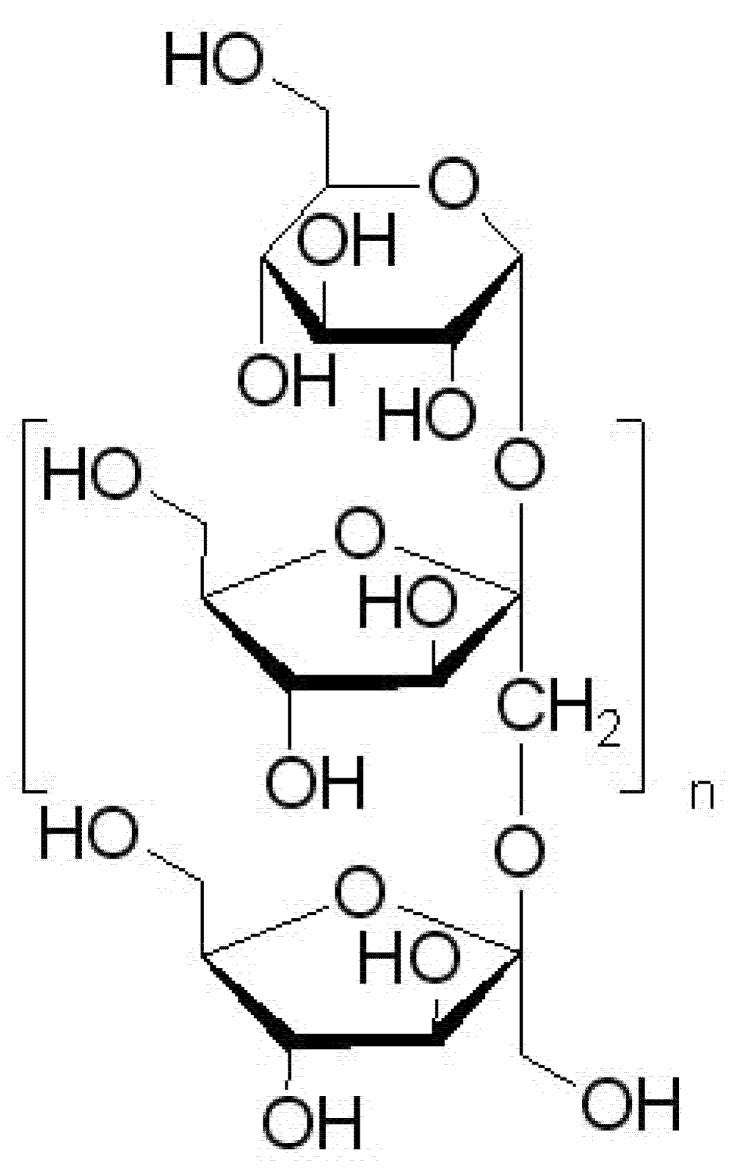
The structural formula of the monomer of the inulin molecule (n = 30–35).

After confirming the potential of using *Cornus mas* fruit, following the assumed research objective, the next stage of the research was to prepare a system based on a prebiotic substance capable of modifying the intestinal microbiome, the effect of which is the long-term control of blood sugar levels. Inulin was chosen as the carrier substance (Figure 6). It is a polysaccharide mainly obtained from chicory, with proven prebiotic potential against *Bifidobacterium* and *Lactobacillus* [67,68,69]. An essential aspect of selecting an appropriate carrier is its safety in DM. Attention should be paid to the glycemic index, which in the case of inulin is relatively low and is in the range of 8–14 [70]. Inulin is a polysaccharide, consisting of monomers linked by β-2,1-glycosidic bonds in an unbranched chain; these bonds cannot be broken down by human digestive enzymes, while the naturally occurring bacterial microflora can decompose it and utilize the resulting simple sugars for their growth [71].

The pre-formulation obtained by suspending fruit extract in the prebiotic carrier was standardized for the content of active substances previously tested in the plant extracts (Table 3), converted into g of the obtained system.

In addition, an activity analysis was performed to control the quality and confirm the activity of the obtained system before proceeding to the next stage of the research (Table 4). The figures showing the activity curves are provided in the Supplementary Materials (Figures S2–S4).

The obtained results indicate high activity of the obtained system; it showed almost ten times higher activity against α-glucosidase than acarbose which is traditionally used in medicine as an inhibitor of carbohydrate digestive enzymes and is also characterized by high antioxidant activity. As part of the preparation, it was possible to preserve the original properties of the plant extracts, enriching them with prebiotic activity.

### 3.3. In Vivo Activity

In the present studies, STZ induced diabetes, reflected in an increase of blood glucose (>270 mg/mL measured 14 and 21 days after STZ injection) as well as a decrease in body-weight (20% vs. control group). *Corni fructus* extract did not influence the measured parameters. The activity of *Corni fructus* in diabetes type I was also evaluated by Gao et al. They revealed that *Corni fructus*-treated diabetic rats showed significant decreases in blood glucose, urinary protein levels, water consumption, and improved lipid profile [72]. The difference between our studies and those mentioned was the time of administration (21 days vs. 40) and dose (50 mg/kg vs. 100, 200 mg/kg). Further studies, using a more extended administration scheme or higher doses of the extract, are required.

### 3.4. Quantitative and Qualitative Analysis of Changes in the Intestinal Microflora

Four feces samples of 10 rats in each group were analyzed at four measuring points (t0, t7 day, t14 day, t21 day). The samples were designated as STZ + extract with inulin, Healthy + extract with inulin, STZ + saline, and Healthy + saline. In all cases studied, the microbiological profile of the intestinal microorganism was similar at a given measuring point; the results are shown in Table 5.

The analyzed groups of microorganisms were divided into three main groups; microorganisms with protective functions (*Bifidobacterium*, *Lactobacillus* sp.), immunostimulatory species (*E. coli*, *Enterococcus* sp.), and proteolytic species (*Proteus* sp., *Pseudomonas* sp., *E. coli* Biovare, *Clostridium* pp.).

The total number of microorganisms in all tested samples ranged from 10^10^–10^12^ CFU/g of feces, with the lowest on the seventh day of feeding. The number of *Bifidobacterium* and *Lactobacillus* bacteria, particularly their quantitative ratio, plays a crucial role in assessing intestinal microbial microbes’ effect. The highest dynamics of *Bifidobacterium* increase were observed in the Healthy + extract with inulin variant. During the 21 days of the experiment, this group of microorganisms increased from 6.7 × 10^6^ CFU/g to 3.4 × 10^8^ CFU/g. In contrast, in the Healthy + saline variant, there was no reduction in *Bifidobacterium* abundance or increase. The value was constant at 10^6^ CFU/g. In the same variant, the number of *Lactobacillus* bacteria did not change throughout the entire feeding period, and its value was close to that of *Bifidobacterium*. The increase in *Lactobacillus* sp. counts was demonstrated in the STZ + extract with inulin variant. In this case, the number of bacteria increased from 5.0 × 10^7^ CFU/g before feeding to 6.4 × 10^8^ CFU/g after 21 days of feeding. In this case, a very considerable reduction in anaerobic bacteria of the genus *Clostridium* sp. from 4.0 × 10^8^ CFU/g to 1.2 × 10^4^ CFU/g was also demonstrated. However, in the Healthy + saline variant, this group of microorganisms remained stable and was initially high. The increase in the number of probiotic bacteria of the genus *Bifidobacterium* sp. and bacteria of the genus *Lactobacillus* sp. was observed in two variants (“STZ + extract with inulin” and “Healthy + extract with inulin”). The probable cause of the growth of these probiotic microorganisms was the addition of inulin. Inulin is a widely used fiber that is considered a prebiotic because of its ability to be selectively used by the gut microbiota for health benefits.

The presence was not demonstrated of yeast-like fungi of the genus Candida in variants I and II. Simultaneously, in III and IV, the number increased from 7 to 21 days of feeding. This indicates the protective properties of the obtained mixture of *Cornus mas* fruit extract with inulin and confirms the lack of relationship between dietary insulin and *Candida* sp. development. Many microorganisms of the genera *Pseudomonas* and *Proteus* were found in none of the examined variants.

## 4. Conclusions

Considering reports in the literature indicating the potential for using *Cornus mas* fruit, studies were carried out to determine the activity profile of different cultivars. The obtained water extracts were standardized for anthocyanins, such as pelargonidin 3-*O*-glucoside and cyanidin-3-*O*-glucoside, loganic acid, and phenolic compounds. As part of the evaluation of the activity profile, inhibition of α-glucosidase was performed. The *Wydubieckij* cultivar (IC_50_ = 45.23 µg/mL) showed 30 times higher activity than the standard acarbose (IC_50_ = 1.22 mg/mL), which was correlated with the content of TPC. In addition, the most potent antioxidant activity was found using water extract from the *Corni fructus* cultivar *Słowianin* with IC_50_ = 343.63 µg/mL and IC_0.5_ = 0.25 µg/mL, respectively, in the DPPH and FRAP methods.

Due to high biological activity and good antioxidant activity, the *Wydubieckij* cultivar was selected for further research. Considering the latest reports on the gut microbiome’s influence on the development and course of DM2, further studies involved development of a pre-formulation containing inulin as a carrier with prebiotic potential. In order to assess the activity of the obtained pre-formulation, it was tested in an in vivo model on rats.

Although we did not observe a decrease in glucose blood level in STZ-treated diabetic rats, we are aware of the limitations of the present studies. Further in vivo research is required to consider different diabetes induction patterns and to adjust the therapeutic dose.

In addition, as part of the control of the activity of the obtained system, fecal analysis was performed to estimate quantitative and qualitative changes in the intestinal microbiome. The obtained results indicated a positive development of prebiotic microorganisms and inhibition of the growth of potentially pathogenic microorganisms.

The obtained results suggest that *Corni fructus* is a potentially valuable raw material for the prophylaxis of symptoms for type I diabetes. Combining it with inulin increases this potential by influencing the intestinal microbiome. Therefore, the tested plant material should be considered a valuable functional food component.

## Figures and Tables

**Table 1:** The content of active compounds in the lyophilized *Cornus mas* fruit.

Cultivar	Content			
	mg GAE/g	mg/g		
	TPC	Loganic Acid	Pelargonidin 3-*O*-Glucoside	Cyanidin-3-*O*-Glucoside
*Bolestraszycki*	8.94 ± 0.83 *	5.38 ± 0.52 *	2.21 ± 0.19 *	1.08 ± 0.13 *
*Florianka*	7.34 ± 0.66 *	4.71 ± 0.45 *	3.09 ± 0.25 *	0.05 ± 0.00 *
*Słowianin*	8.42 ± 0.79 *	4.96 ± 0.12 *	2.28 ± 0.11 *	0.94 ± 0.09 *
*Wydubieckij*	16.03 ± 1.28 *	2.82 ± 0.27 *	0.10 ± 0.02 *	0.10 ± 0.00 *

Data expressed as mean ± SD; * significance with *p* ≤ 0.05.

**Table 2:** In vitro activity of the lyophilized *Cornus mas* fruit water extracts.

Cultivar	IC_50_ (µg/mL)		IC_0.5_ (µg/mL)
	Inhibition of α-Glucosidase	DPPH	FRAP
*Bolestraszycki*	167.94 ± 2.97 *	383.49 ± 1.54 *	0.30 ± 0.02 *
*Florianka*	178.26 ± 3.64 *	471.25 ± 5.20 *	0.32 ± 0.02 *
*Słowianin*	170.47 ± 4.92 *	343.63 ± 1.29 *	0.25 ± 0.01 *
*Wydubieckij*	45.23 ± 1.96 *	417.56 ± 0.81 *	0.31 ± 0.02 *

Data expressed as mean ± SD; * significance with *p* ≤ 0.05.

**Table 3:** The content of active compounds in the lyophilized *Cornus mas* system.

	Content			
	mg GAE/g	mg/g		
	TPC	Loganic Acid	Pelargonidin 3-*O*-Glucoside	Cyanidin-3-*O*-Glucoside
Prebiotic system	119.0 ± 2.34	5.33 ± 0.48	1.30 ± 0.11	0.32 ± 0.05

Data expressed as mean ± SD.

**Table 4:** In vitro activity of the lyophilized *Cornus mas* fruit system.

Cultivar	IC_50_ (mg/mL)		IC_0.5_ (mg/mL)
	Inhibition of α-Glucosidase	DPPH	FRAP
Prebiotic system	0.173 ± 0.025	1.176 ± 0.066	0.905 ± 0.07

Data expressed as mean ± SD.

**Table 5:** Effect of *Cornus mas* fruit with inulin on the microbiological profile of the intestinal microorganisms in rats.

**STZ + Extract with Inulin**	**T0**	**T7**	**T14**	**T21**
	**CFU/g**			
Total number of microorganisms	5.00 × 10^11^	1.90 × 10^10^	6.70 × 10^11^	7.60 × 10^12^
*Bifidobacterium*	6.70 × 10^6^	2.30 × 10^6^	7.70 × 10^7^	2.30 × 10^7^
*Lactobacillus*	5.00 × 10^7^	4.50 × 10^7^	5.40 × 10^8^	6.40 × 10^8^
*Enterococcus*	1.20 × 10^6^	7.60 × 10^7^	6.50 × 10^8^	4.30 × 10^8^
*E. coli*	1.00 × 10^3^	5.40 × 10^5^	3.20 × 10^5^	1.30 × 10^6^
*E. coli Biovare*	3.10 × 10^5^	2.30 × 10^5^	3.20 × 10^4^	3.20 × 10^4^
*Clostridium*	4.00 × 10^8^	6.70 × 10^5^	3.20 × 10^5^	1.20 × 10^4^
*Proteus*	<10	<10	<10	<10
*Pseudomonas*	<10	<10	<10	<10
*Candida*	<10	<10	<10	<10
**Healthy + Extract with Inulin**	**T0**	**T7**	**T14**	**T21**
	**CFU/g**			
Total number of microorganisms	5.00 × 10^11^	5.60 × 10^10^	5.60 × 10^12^	9.40 × 10^11^
*Bifidobacterium*	6.70 × 10^6^	6.50 × 10^7^	5.40 × 10^7^	3.40 × 10^8^
*Lactobacillus*	5.00 × 10^7^	1.90 × 10^7^	7.60 × 10^7^	2.30 × 10^7^
*Enterococcus*	1.20 × 10^6^	6.60 × 10^6^	2.30 × 10^8^	5.40 × 10^8^
*E. coli*	1.00 × 10^3^	5.40 × 10^4^	5.40 × 10^4^	3.40 × 10^4^
*E. coli Biovare*	3.10 × 10^5^	3.20 × 10^4^	2.10 × 10^4^	3.20 × 10^4^
*Clostridium*	4.00 × 10^8^	4.50 × 10^7^	3.50 × 10^5^	8.70 × 10^5^
*Proteus*	<10	<10	<10	<10
*Pseudomonas*	<10	<10	<10	<10
*Candida*	<10	<10	<10	<10
**STZ + Saline**	**T0**	**T7**	**T14**	**T21**
	**CFU/g**			
Total number of microorganisms	5.00 × 10^11^	4.30 × 10^10^	5.40 × 10^11^	2.30 × 10^11^
*Bifidobacterium*	6.70 × 10^6^	4.50 × 10^6^	4.50 × 10^6^	7.60 × 10^6^
*Lactobacillus*	5.00 × 10^7^	5.40 × 10^6^	3.30 × 10^7^	6.70 × 10^6^
*Enterococcus*	1.20 × 10^6^	5.20 × 10^6^	3.20 × 10^7^	4.50 × 10^7^
*E. coli*	1.00 × 10^3^	2.20 × 10^6^	1.20 × 10^7^	8.90 × 10^7^
*E. coli Biovare*	3.10 × 10^5^	2.30 × 10^3^	2.50 × 10^4^	2.30 × 10^5^
*Clostridium*	4.00 × 10^8^	1.50 × 10^7^	2.30 × 10^7^	2.30 × 10^5^
*Proteus*	<10	<10	<10	<10
*Pseudomonas*	<10	<10	<10	<10
*Candida*	<10	2.20 × 10^3^	2.30 × 10^4^	2.10 × 10^4^
**Healthy + Saline**	**T0**	**T7**	**T14**	**T21**
	**CFU/g**			
Total number of microorganisms	5.00 × 10^11^	1.40 × 10^10^	2.30 × 10^10^	4.30 × 10^10^
*Bifidobacterium*	6.70 × 10^6^	4.50 × 10^6^	1.20 × 10^6^	2.40 × 10^5^
*Lactobacillus*	5.00 × 10^7^	4.50 × 10^6^	4.50 × 10^6^	3.80 × 10^6^
*Enterococcus*	1.20 × 10^6^	2.30 × 10^5^	2.30 × 10^5^	4.50 × 10^5^
*E. coli*	1.00 × 10^3^	1.20 × 10^4^	4.30 × 10^4^	1.30 × 10^5^
*E. coli Biovare*	3.10 × 10^5^	1.80 × 10^4^	4.50 × 10^4^	8.40 × 10^4^
*Clostridium*	4.00 × 10^8^	3.40 × 10^8^	4.50 × 10^7^	9.80 × 10^8^
*Proteus*	<10	<10	<10	<10
*Pseudomonas*	<10	<10	<10	<10
*Candida*	<10	4.50 × 10^2^	5.20 × 10^3^	7.60 × 10^3^

## Data Availability

The data is contained within the article or Supplementary Material.

## Supplementary Materials

The following are available online at https://www.mdpi.com/article/10.3390/antiox11020380/s1, Figure S1. Chromatogram showing the loganic acid pattern in the developed method; Table S1. Statistical assay of linear plots of the anthocyanin determined by the UHPLC-DAD method; Table S2. Statistical assay of linear plots of the loganic acid determined by the UHPLC-DAD method; Table S3. The content of active compounds in the lyophilized *Cornus mas* fruit in 70% EtOH extract; Table S4. Water content in freeze-dried fruit expressed as % of weight loss during drying in a moisture analyzer; Figure S2. The α-glucosidase inhibitory activity of *Corni fructus* prebiotic system; Figure S3. Antioxidant activity of *Corni fructus* system by DPPH assay; Figure S4. Antioxidant activity of *Corni fructus* system by FRAP assay; Figure S5. Curve for gallic acid used to calculate TPC content.
