# Supplementary material for: Potential for Prebiotic Stabilized Cornus mas L. Lyophilized Extract in the Prophylaxis of Diabetes Mellitus in Streptozotocin Diabetic Rats
Source: Antioxidants (Basel). 2022 Feb 14;11(2):380. doi: 10.3390/antiox11020380 (PMC8868578; doi:10.3390/antiox11020380)
Supplement: Supplementary file 1 [file antioxidants-11-00380-s001.zip › antioxidants-1576143-supplementary.pdf]

antioxidants

Supplementary materials

Potential for the Prebiotic Stabilized *Cornus mas* L. Lyophilized Extract in the Prophylaxis of Type II Diabetes

Szymon Sip [1], Justyna Chanaj-Kaczmarek [1],Daria Szymanowska [2], Krystyna Skalicka-Woźniak[3], Barbara Budzyńska [4], Olga Wronikowska-Denysiuk [4], Tymoteusz Słowik [5], Piotr Szulc[6], Judyta Cielecka-Piontek [1*]

[1] Department of Pharmacognosy, Poznan University of Medical Sciences, 4 Swiecickiego Street, 60-781 Poznan, Poland; szymonsip@ump.edu.pl (S.S.), justyna.chanaj-kaczmarek@ump.edu.pl (J.C-K.), jpiontek@ump.edu.pl (J.C.-P.)
[2] Poznan University of Life Sciences, 31 Wojska Polskiego Street, 60-634 Poznan, Poland; darszy@up.poznan.pl (D.S)
[3] Department of Natural Products Chemistry, Medical University of Lublin, 1 Chodźki Street, 20-093 Lublin, Poland; kskalicka@pharmacognosy.org (K.S.-W.)
[4] Independent Laboratory of Behavioral Studies, Medical University of Lublin, Lublin, Poland; barbara.budzynska@umlub.pl (B.B.), olga.wronikowska@gmail.com (O.W.-D.)
[5] Experimental Medicine Center, Medical University of Lublin, Jaczewskiego 8D, 20-090 Lublin, Poland; Tymoteusz.slowik@o2.pl (T.S.)
[5] Department of Agronomy, Poznań University of Life Sciences, Dojazd 11, 60-632 Poznań, Poland; piotr.szulc@up.poznan.pl (P.S.)
* Corresponding author: jpiontek@ump.edu.pl

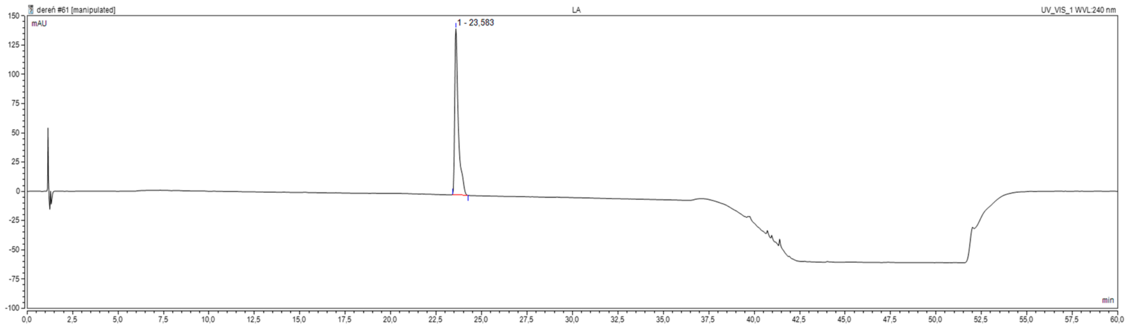

Figure S1. Chromatogram showing the loganic acid pattern in the developed method.

Table S1. Statistical assay of linear plots of the anthocyanin determined by the UHPLC-DAD method

Parameter	Delphinidin 3-*O*-glucoside	Cyanidin 3-*O*-glucoside	Pelargonidin 3-*O*-glucoside
Linearity: y = ax + b			
a ± S_a	0.2712 ± 0.0140	0.3384 ± 0.0082	0.2460 ± 0.0040
b ± S_b	7.2327± 0.9850	8.8446 ± 0.2884	14.7807 ± 0.2817
correlation coefficient (r)	0.9973	0.9994	0.9997
Limit of detection (LOD): LOD = 3 SD/a (µg mL^{-1})	9.17	2.15	2.89
Limit of quantification (LOQ): LOQ = 10 SD/a (µg mL^{-1})	27.80	6.52	8.76
Range of linearity (µg mL^{-1})	30 - 80	15 - 80	30 – 100
Precision, RSD			
	30 (µg mL^{-1}) 1.34	15 (µg mL^{-1}) 0.60	30 (µg mL^{-1}) 0.74
	50 (µg mL^{-1}) 2.31	25 (µg mL^{-1}) 3.04	50 (µg mL^{-1}) 0.27
	80 (µg mL^{-1}) 2.43	40 (µg mL^{-1}) 5.58	80 (µg mL^{-1}) 0.64
Intra-day, RSD			
	30 (µg mL^{-1}) 1.72	30 (µg mL^{-1}) 0.99	30 (µg mL^{-1}) 0.88
	50 (µg mL^{-1}) 1.98	50 (µg mL^{-1}) 1.35	50 (µg mL^{-1}) 0.32
	80 (µg mL^{-1}) 2.25	80 (µg mL^{-1}) 2.44	80 (µg mL^{-1}) 0.75
Retention time (min)	6.16	8.93	12.15

Table S2. Statistical assay of linear plots of the loganic acid determined by the UHPLC-DAD method

Parameter	Loganic acid	
Linearity: y = ax + b		
a ± S_a	183.063 ± 3.469	
b ± S_b	-11.047± 1.226	
correlation coefficient (r)	0.9963	
Limit of detection (LOD): LOD = 3 SD/a (mg mL^{-1})	0,257	
Limit of quantification (LOQ): LOQ = 10 SD/a (mg mL^{-1})	0,848	
Range of linearity (mg mL^{-1})	0,96 – 5,76	
Precision, RSD		
	0,96 (mg mL^{-1})	0,07
	3,84 (mg mL^{-1})	0,12
	5,76 (mg mL^{-1})	0,17
Intra-day, RSD		
	0,96 (mg mL^{-1})	0,09
	3,84 (mg mL^{-1})	0,15
	5,76 (mg mL^{-1})	0,22
Retention time (min)	23.5	

Table S3. The content of active compounds in the lyophilized *Cornus mas* fruit in 70% EtOH extract.

Cultivar	Content			
	mg GAE/g	mg/g		
	TPC	Loganic acid	Pelargonidin 3-O-glucoside	Cyanidin-3-O-glucoside
Bolestraszycki	11.14± 0.77	7.08 ± 0.33	2.68 ± 0.09	1.30 ± 0.12
Florianka	9.51± 0.54	6.08 ± 0.24	3.98 ± 0.11	0.06 ± 0.01
Słowianin	10.65± 0.69	6.50 ± 0.39	2.84 ± 0.10	1.17 ± 0.07
Wydubieckij	19.16± 1.10	3.76 ± 0.19	0.12 ± 0.02	0.13 ± 0.02

Table S4. Water content in freeze-dried fruit expressed as % of weight loss during drying in a moisture analyzer.

Variety	Water content in freeze-dried fruit [%]
Bolestraszycki	6,83%
Florianka	9,60%
Słowianin	5,94%
Wydubiecki	6,63%

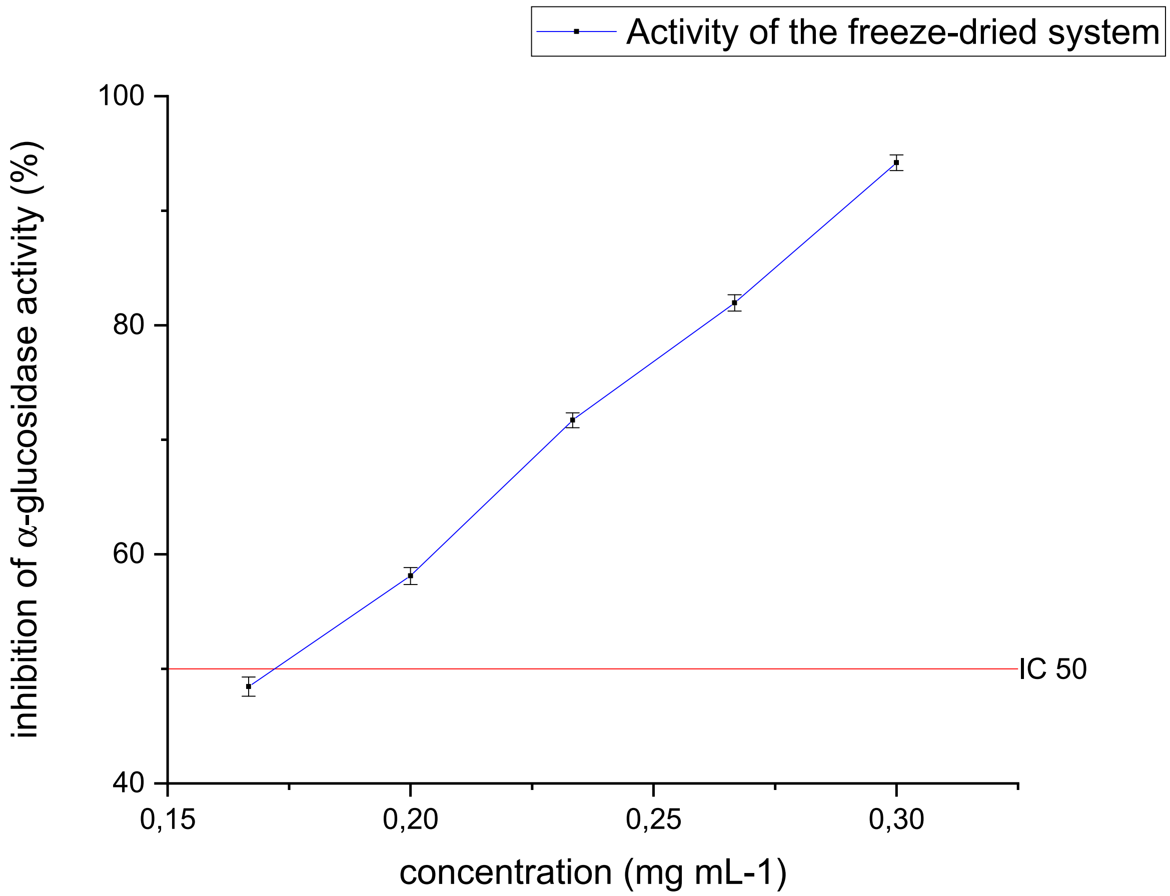

Figure S2. The α-glucosidase inhibitory activity of *Corni fructus* prebiotic system

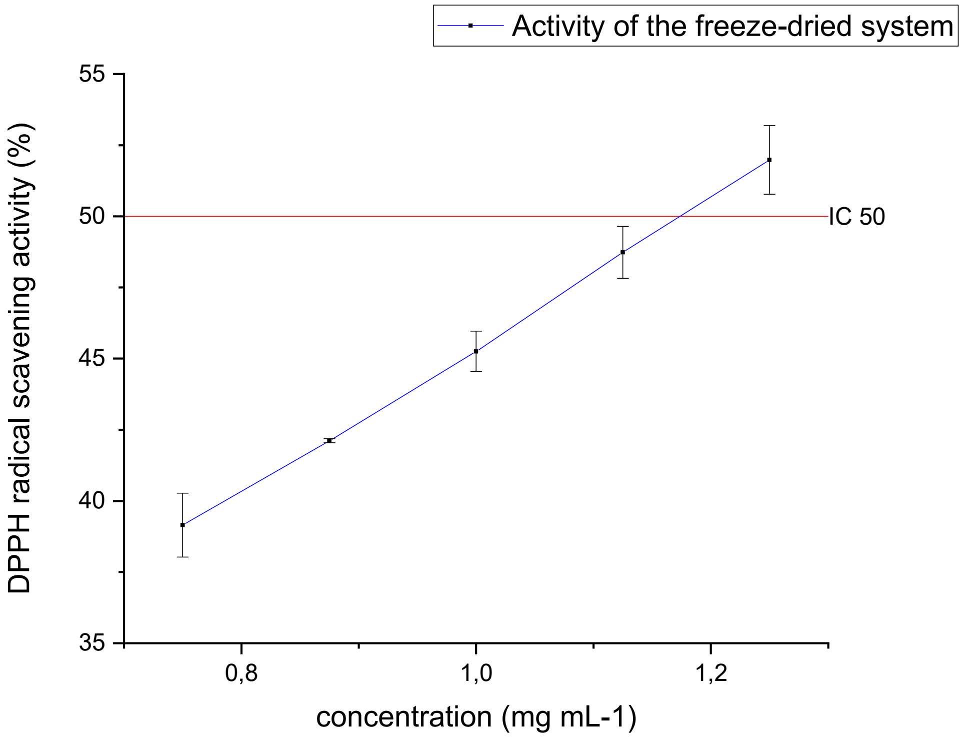

Figure S3. Antioxidant activity of Corni fructus system by DPPH assay

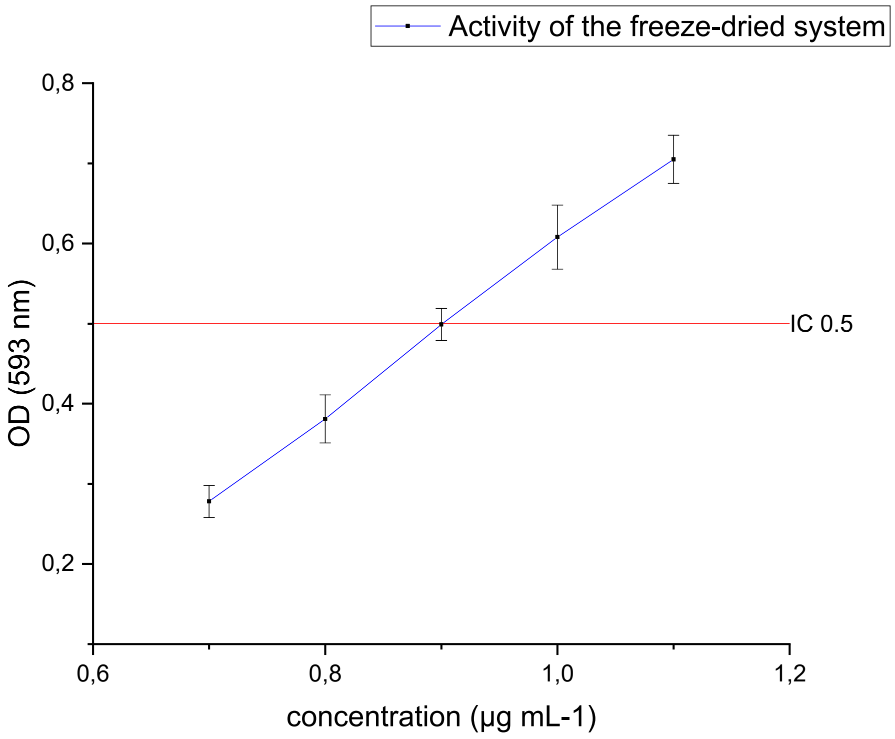

Figure S4. Antioxidant activity of Corni fructus system by FRAP assay

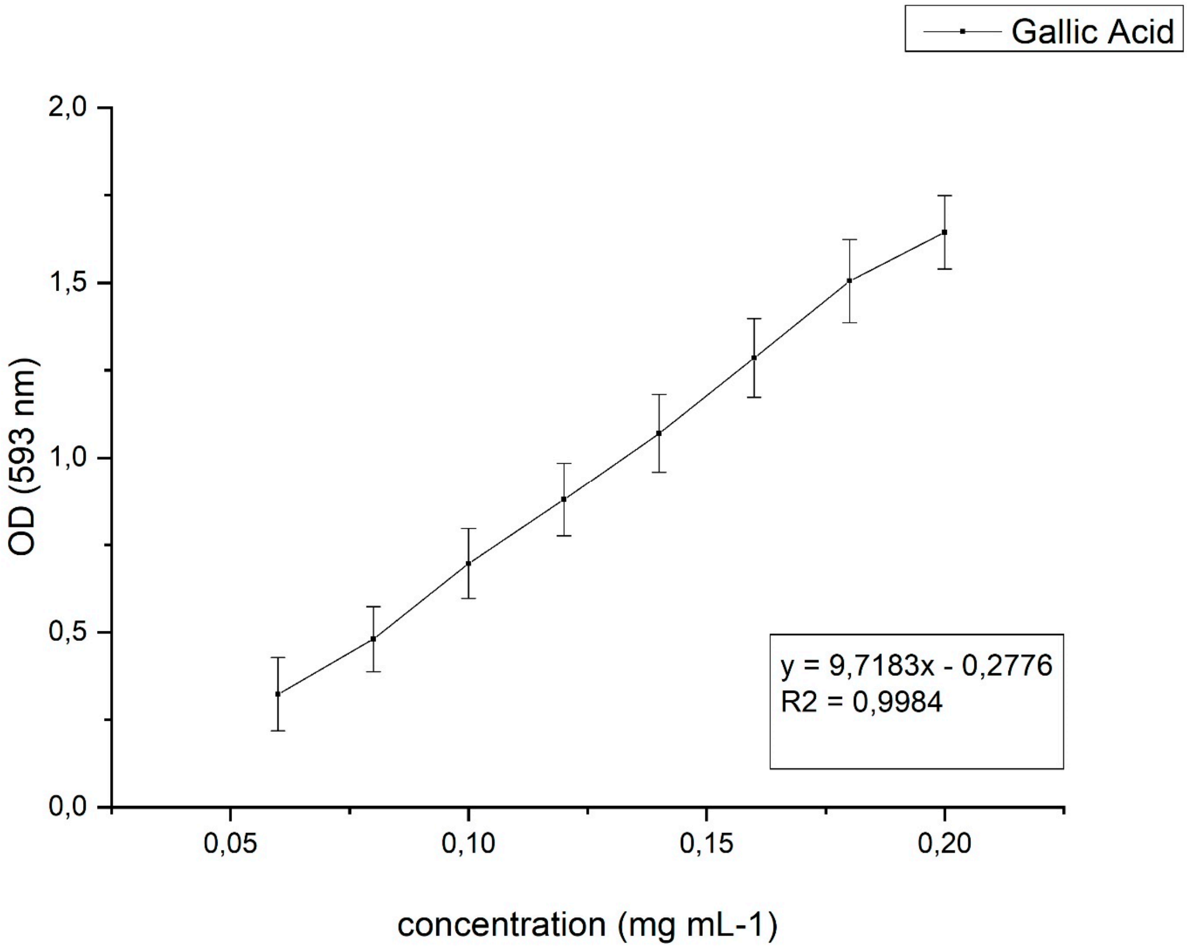

Figure S5. Curve for gallic acid used to calculate TPC content.
